# Nomogram for predicting suicide risk in pancreatic cancer patients: a retrospective study based on SEER database

**DOI:** 10.3389/fpsyg.2025.1500569

**Published:** 2025-07-11

**Authors:** Jinzhe Bi, Yaqun Yu

**Affiliations:** Department of Hepatobiliary and Pancreatic Surgery, Affiliated Hospital of Guilin Medical University, Guilin, China

**Keywords:** pancreatic cancer, suicide, surveillance epidemiology and end results (SEER) database, prediction, nomogram, machine learning, precision medicine

## Abstract

**Background:**

The incidence of suicide among individuals diagnosed with pancreatic cancer surpasses that observed in both the general population and patients with other types of cancer. This study seeks to identify independent risk factors associated with suicide post-pancreatic cancer diagnosis and to develop a predictive model that may contribute to reducing suicide rates in this patient population.

**Methods:**

We screened and identified patients diagnosed with pancreatic cancer from the Surveillance, Epidemiology, and End Results (SEER) database between 2000 and 2020, and randomly divided them into training and validation sets. A combination of least absolute shrinkage and selection operator (LASSO) and Cox proportional hazards regression was employed to identify relevant predictive factors. Based on these analyses, we developed a novel nomogram to visualize suicide risk following a pancreatic cancer diagnosis. The performance of the nomogram was evaluated utilizing the concordance index (C-index), receiver operating characteristic (ROC) curves, calibration curves, and decision curve analysis (DCA).

**Results:**

A cohort of 129,301 eligible pancreatic cancer patients was incorporated into the study. Age at diagnosis, sex, marital status, surgery, and chemotherapy are independent predictors of suicide in pancreatic cancer patients. The accuracy of the nomogram was evaluated using the C-index and ROC curves, demonstrating acceptable performance in both the training and validation sets. Additionally, the calibration plot exhibited a high degree of concordance between the predicted and observed values, while the clinical decision curves indicated a significant clinical benefit.

**Conclusion:**

The proposed nomogram is a reliable tool for predicting suicide risk following a pancreatic cancer diagnosis, providing valuable support for personalized and precise risk quantification in clinical practice.

## Introduction

Suicide is the intentional act of ending one's life, a deeply tragic event that affects not only individuals and families but also entire communities and nations, creating a significant burden on public health resources. Suicide is the tenth leading cause of death among people of all ages in the United States (Fazel et al., 2020). According to estimates from the World Health Organization (WHO) and the Global Burden of Disease (GBD) study, approximately 800,000 people die by suicide each year (Heinrich et al., 2022; GBD 2017 Causes of Death Collaborators, 2018). Cancer is a significant global health challenge and one of the leading causes of death worldwide, claiming over 10 million lives annually (Zaimy et al., 2017). In the United States, cancer ranks as the second leading cause of death across all age groups (Siegel et al., 2024). Projections estimate that by 2040, the number of new cancer cases will rise to 28.4 million, imposing a significant economic, healthcare, and financial burden on society (Sung et al., 2021). Increasing evidence indicates that cancer patients have a higher risk of suicide compared to the general population (O'Rourke, 2022; Misono et al., 2008; Choi and Park, 2020). Cancer patients have an increased risk of suicide, primarily driven by psychological distress, treatment-related side effects, a decline in quality of life, and severe, uncontrolled cancer-related pain (Heinrich et al., 2022; Zaorsky et al., 2019; Das, 2019). The combination of physical and emotional suffering can be overwhelming, further intensifying feelings of helplessness and despair, leading to the development of depression and anxiety, and ultimately increasing the risk of suicide in cancer patients (Hofmann et al., 2023; Ribeiro et al., 2018; Henson et al., 2019). Therefore, identifying which subgroups of cancer patients are at a higher risk of suicide could be beneficial.

Pancreatic cancer is one of the most aggressive malignancies, characterized by difficult early diagnosis and poor prognosis, with a 5-year survival rate of only 13%, and is projected become the third-leading cause of cancer-related death in 2024 (Siegel et al., 2024). Pancreatic cancer patients are more vulnerable to severe mental health disorders, especially depression and anxiety, compared to those with other solid tumors (Dengsø et al., 2020; Holland et al., 1986). It is noteworthy that pancreatic cancer has been reported to have the highest suicide rate within the 1st year after diagnosis compared to other cancers (Saad et al., 2019). However, there is currently limited research on suicide among pancreatic cancer patients, and reliable long-term predictive models for assessing individual suicide risk are lacking. Machine learning offers more accurate and reliable predictions and assessments of suicide risk compared to traditional methods (Lejeune et al., 2022). A robust predictive model can accurately assess the suicide risk for individual patients, thereby enabling clinicians to efficiently identify those at high risk and expedite their referral to mental health professionals for timely intervention, ultimately contributing to a reduction in suicide rates. In a nomogram, complex statistical prediction models are streamlined into a single numerical estimate, which accurately conveys the probability of an event, taking into account the unique conditions of each individual (Jeong et al., 2020; Balachandran et al., 2015). This approach is highly useful for practical application in clinical settings to predict the likelihood of suicide in pancreatic cancer patients. In this study, we analyzed the Surveillance, Epidemiology, and End Results (SEER) database to identify independent risk factors for suicide in pancreatic cancer patients. Additionally, we developed and validated a nomogram to predict the suicide risk in pancreatic cancer patients.

## Patients and methods

### Patient selection and variables

**Figure 1:**
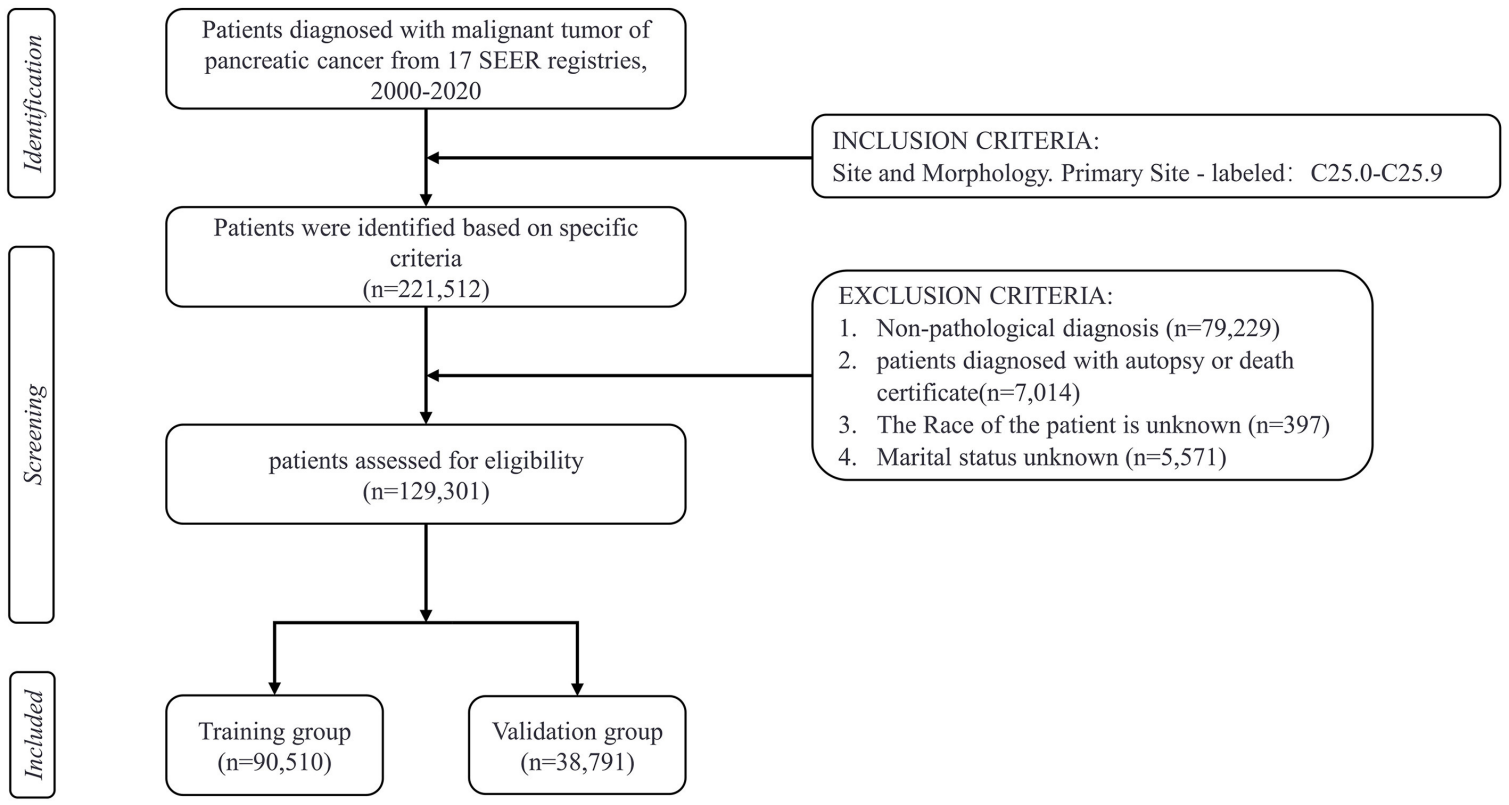
Flowchart depicting the process of patient selection.

This study is an observational retrospective cohort study. The current research data on pancreatic cancer patients is extracted from the SEER database from 2000 to 2020, which includes data from 17 cancer registries. The SEER program, initiated in 1973 and backed by the National Cancer Institute (NCI) in the United States, compiles data on the incidence and survival characteristics of malignant tumors, covering about 28% of the population through cancer registries across the country (Cronin et al., 2014). The data used in this study were obtained using SEER^*^Stat software (version 8.4.3) in August 2024. The inclusion criteria consisted of the following: (1) Patients with malignant pancreatic tumors were classified under primary site codes C25.0 to C25.9, according to the International Classification of Diseases for Oncology, 3rd Edition (ICD-O-3; Gordon-Dseagu et al., 2018). (2) Patient demographic variables, including age at diagnosis (<65, ≥ 65 years old), sex, race (white, black, Asian, other), marital status, annual household income, and location of residence, were available. (3) Clinicopathological information, including histological grade, stage (localized, regional, distant, unknown/other), primary tumor site (head, body or tails, other/unknown), histological type, surgery, radiotherapy, and chemotherapy. The exclusion criteria during the patient selection process were as follows: patients with non-pathological diagnosis, unknown marital status, unknown race, unknown survival time, incomplete medical records, and diagnosed with pancreatic cancer only by autopsy or death certificate. The primary outcome of concern was suicide deaths after a diagnosis of pancreatic cancer, with the cause of death documented in the SEER database as “Suicide and Self-Inflicted Injury.” Marital status was categorized as married, unmarried, separated, divorced, or widowed (SDW). According to the Rural-Urban Continuum Codes for 2023, place of residence is either metropolitan county, non-metropolitan or unknown (U.S. Department of Agriculture, 2023). The histological classifications included pancreatic ductal adenocarcinoma (PDAC), pancreatic cystic neoplasm (PCN), pancreatic neuroendocrine tumor (pNET), as well as unknown or other types (Haeberle and Esposito, 2019). A total of 129,301 eligible patients were ultimately included in the study, with the detailed screening process shown in Figure 1. Since SEER data is publicly available and contains no identifiable information or personal details, ethical review or informed consent is not required.

### Statistical analyses

In this research, all statistical analyses were performed using SPSS software (version 26.0) and R software (R Foundation, Vienna, Austria, version 4.2.3, http://www.r-project.org). Continuous variables following a normal distribution were reported as the mean and standard deviation (SD), whereas non-normally distributed variables were expressed as the median and interquartile range (IQR). Comparisons of continuous variables were made using the Student's *t*-test for normally distributed data, and the Mann-Whitney U test for non-normally distributed data. Categorical variables were presented as frequencies and percentages (%), with comparisons conducted using either Fisher's exact test or Pearson's chi-square test. A *P*-value of < 0.05 (two-sided) was considered statistically significant. The dataset was divided into training and validation sets in a 7:3 ratio through stratified random sampling with the createDataPartition () function in the “caret” package. The nomogram was constructed using the training set, and its performance was evaluated with the validation set. The least absolute shrinkage and selection operator (LASSO) is a regularization technique in regression that introduces a penalty to promote sparsity, enabling variable selection and model simplification while preserving high predictive accuracy (Jacobucci et al., 2016). Cox proportional hazards regression is a widely utilized method in survival analysis. We performed LASSO regression using the “glmnet” package, and Cox proportional hazards regression was conducted using the “survival” package. We integrated LASSO with multivariable Cox proportional hazards regression to identify independent risk factors, leveraging both the regularization of LASSO and the robustness of Cox regression in handling survival data and time-dependent events, thereby ensuring stable variable selection and enhancing the generalizability of the model, a strategy widely employed in oncology prognostic research (Lu et al., 2025; Zhou et al., 2021). The final predictive nomogram was constructed and visualized with the “rms” package. The performance of the nomogram was evaluated by the concordance index (C-index) calculated with the “rms” package, the calibration curves generated using the calibration function in the “rms” package, and decision curve analysis (DCA) performed with the “ggDCA” and “ggscidca” packages. Additionally, time-dependent receiver operating characteristic (ROC) curves were generated using the “timeROC” and “survivalROC” packages to evaluate the predictive performance of the nomogram at various time points, with the area under the curve (AUC) calculated for each. In accordance with the median risk score, patients were stratified into high-risk and low-risk groups. Kaplan-Meier (K-M) curves were employed to depict the differences in suicide-specific survival probabilities over time between the two groups, and the log-rank test was applied to assess statistical significance.

## Results

### Baseline clinical characteristics of patients

**Figure 2:**
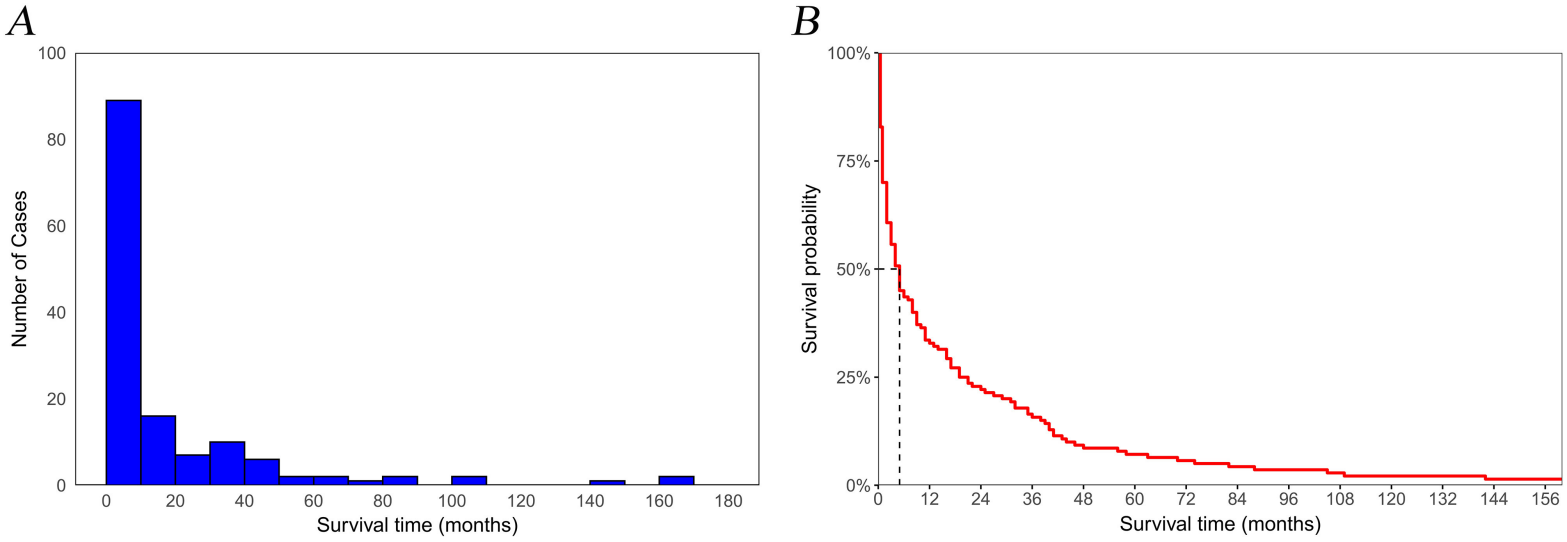
Temporal trends in suicide among pancreatic cancer patients. **(A)** number of cases; **(B)** survival rates.

In this study, a cohort of 129,301 patients diagnosed with pancreatic cancer was included, with 90,510 patients allocated to the training set and 38,791 to the validation set for analysis. Within the study cohort, 140 patients (0.1%) succumbed to suicide. A total of 79,804 patients (61.7%) were aged 65 years or older, comprising 67,625 men and 61,676 women, yielding a male-to-female ratio of 1:1.09. The predominant racial demographic was White, accounting for 80.1% of the cohort. Additionally, 77,385 patients (59.8%) were married. The annual income of 51.4% of the patients ranged between $45,000 to $74,999, and 58.7% resided in metropolitan areas. Regarding clinicopathological features, 54.4% of the patients presented with distant metastasis at the time of diagnosis, and 47.4% of the tumors were situated in the head of the pancreas. In terms of treatment modalities, 37,347 patients (28.9%) underwent surgical intervention, 18,983 patients (14.7%) received radiotherapy, and 67,319 patients (52.1%) were administered chemotherapy. The overall median survival was 6 months, while the median survival for the suicide population was 4 months (Figure 2). No statistically significant differences in demographic and clinicopathological characteristics were identified between the training and validation set (all P > 0.05; Table 1).

**Table 1:** Baseline clinical characteristics of patients diagnosed as pancreatic cancer.

**Characteristic**	**Training group**	**Validation group**	**Overall**	***P*-value**
	**(*****n*** = **90,510)**, ***n*** **(%)**	**(*****n*** = **38,791)**, ***n*** **(%)**	**(*****n*** = **129,301)**, ***n*** **(%)**	
**Year of diagnosis**				0.934
2000–2006	22,733 (25.1)	9,696 (25.0)	32,429 (25.1)	
2007–2012	24,696 (27.3)	10,524 (27.1)	35,220 (27.2)	
2013–2020	43,081 (47.6)	18,571 (47.9)	61,652 (47.7)	
**Age at diagnosis, years**				0.507
<65	34,741 (38.4)	14,756 (38.0)	49,497 (38.3)	
≥65	55,769 (61.6)	24,035 (62.0)	79,804 (61.7)	
**Sex**				0.983
Female	43,188 (47.7)	18,488 (47.7)	61,676 (47.7)	
Male	47,322 (52.3)	20,303 (52.3)	67,625 (52.3)	
**Race**				0.922
White	72,522 (80.1)	31,080 (80.1)	103,602 (80.1)	
Black	10,146 (11.2)	4,402 (11.3)	14,548 (11.3)	
Asian	7,048 (7.8)	2,994 (7.7)	10,042 (7.8)	
Other	794 (0.9)	315 (0.8)	1,109 (0.9)	
**Marital status**				0.969
Married	54,120 (59.8)	23,265 (60.0)	77,385 (59.8)	
SDW	23,509 (26.0)	10,000 (25.8)	33,509 (25.9)	
Unmarried	12,881 (14.2)	5,526 (14.2)	18,407 (14.2)	
**Grade**				0.642
Grade I/II	19,823 (21.9)	8,434 (21.7)	28,257 (21.9)	
Grade III/IV	13,770 (15.2)	5,794 (14.9)	19,564 (15.1)	
Unknown	56,917 (62.9)	24,563 (63.3)	81,480 (63.0)	
**Stage**				0.789
Localized	9,587 (10.6)	4,076 (10.5)	13,663 (10.6)	
Regional	28,452 (31.4)	12,184 (31.4)	40,636 (31.4)	
Distant	49,124 (54.3)	21,168 (54.6)	70,292 (54.4)	
Unknown/unstaged	3347 (3.7)	1,363 (3.5)	4,710 (3.6)	
**Primary site**				0.821
Head	42,843 (47.3)	18,492 (47.7)	61,335 (47.4)	
Body/tail	25,213 (27.9)	10,784 (27.8)	35,997 (27.8)	
Other/unknown	22,454 (24.8)	9,515 (24.5)	31,969 (24.7)	
**Histological type**				0.942
PDAC	69,664 (77.0)	29,881 (77.0)	99,545 (77.0)	
PCN	4,685 (5.2)	2,037 (5.3)	6,722 (5.2)	
pNET	6,473 (7.2)	2,701 (7.0)	9,174 (7.1)	
Unknown/Other	9,688 (10.7)	4,172 (10.8)	13,860 (10.7)	
**Surgery**				0.69
No/unknown	64,303 (71.0)	27,651 (71.3)	91,954 (71.1)	
Yes	26,207 (29.0)	11,140 (28.7)	37,347 (28.9)	
**Radiotherapy**				0.148
No/unknown	77,336 (85.4)	32,982 (85.0)	110,318 (85.3)	
Yes	13,174 (14.6)	5,809 (15.0)	18,983 (14.7)	
**Chemotherapy**				0.76
No/unknown	43,448 (48.0)	18,534 (47.8)	61,982 (47.9)	
Yes	47,062 (52.0)	20,257 (52.2)	67,319 (52.1)	
**Status**				0.934
None suicide	90,414 (99.9)	38,747 (99.9)	129,161 (99.9)	
Suicide	96 (0.1)	44 (0.1)	140 (0.1)	
**Annual household income**				0.997
<$45,000	4,620 (5.1)	1,998 (5.2)	6,618 (5.1)	
$45,000–$74,999	46,559 (51.4)	19,966 (51.5)	66,525 (51.4)	
>$75,000	39,331 (43.5)	16,827 (43.4)	56,158 (43.4)	
**Residence**				0.327
Metropolitan	53,030 (58.6)	22,901 (59.0)	75,931 (58.7)	
Non-Metro/unknown	37,480 (41.4)	15,890 (41.0)	53,370 (41.3)	
**Survival months (P**_**25**_ **, P**_**75**_ **)**	6 (2,16)	6 (2,16)	6 (2,16)	0.131

SDW, Separated + Divorced + Widowed.

### Feature selection

**Figure 3:**
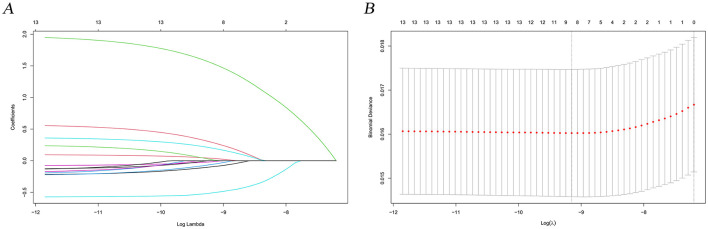
LASSO regression analysis for predictive factor selection. **(A)** variable coefficient diagram of lasso regression model; **(B)** lasso cross-validation curve.

**Figure 4:**
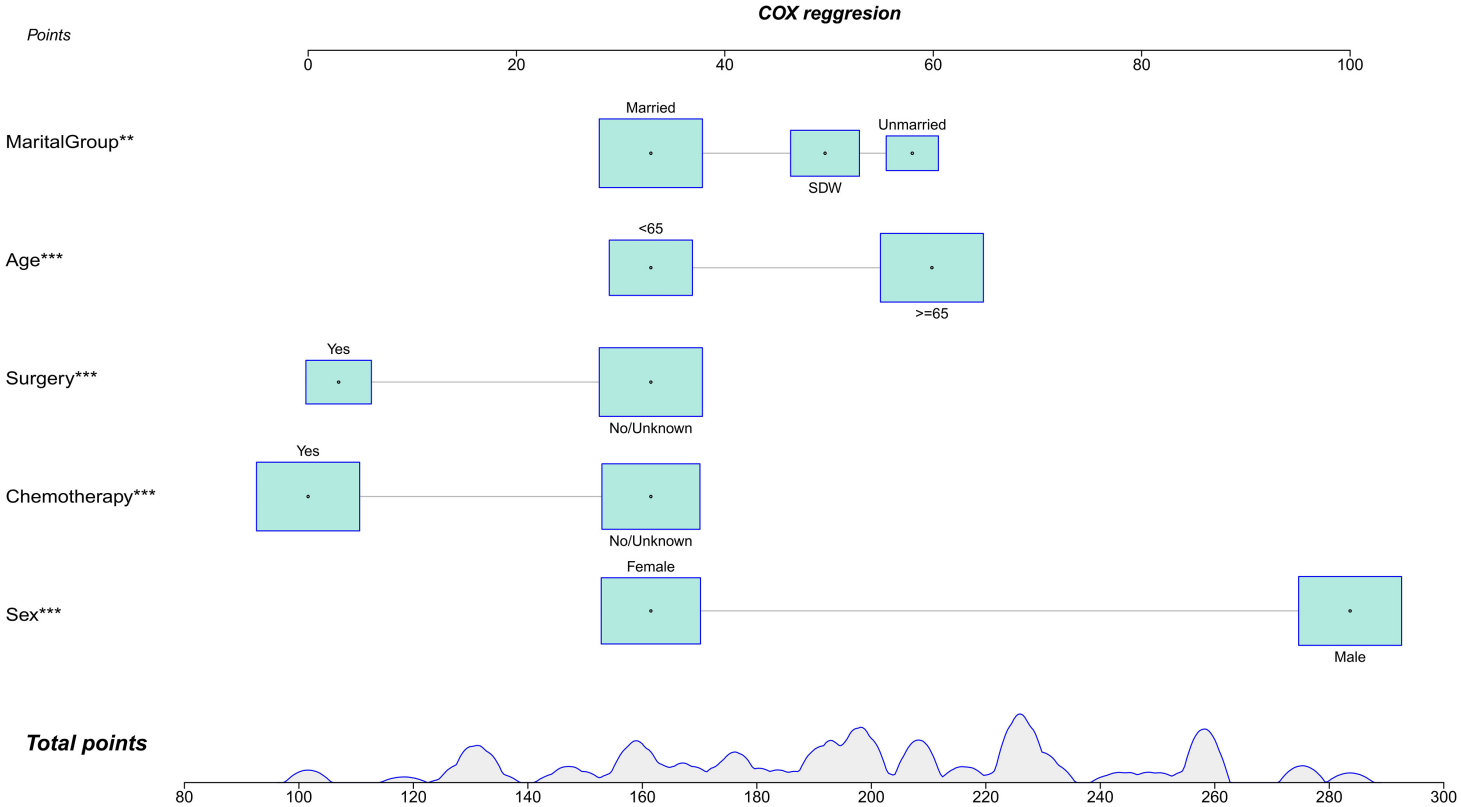
Nomogra for predicting the risk of suicide in individuals with pancreatic cancer. **P < 0.01; ***P < 0.001.

In this study, lambda.min was identified as the optimal value, as shown in Figures 3A, B. The variables chosen through LASSO regression were age at diagnosis, sex, marital status, surgery and chemotherapy. The results of the univariate and multivariate Cox proportional hazards regression models for suicide risk in the training set are displayed in Table 2. The findings reveal that age 65 or older (HR = 2.295; 95% CI: 1.450–3.630, *P* < 0.001), male gender (HR = 7.902; 95% CI: 4.256–14.673, *P* < 0.001), and unmarried status (HR = 2.159; 95% CI: 1.292–3.611, *P* = 0.003, compared to married), undergoing surgery (HR = 0.403; 95% CI: 0.251–0.647, *P* < 0.001), and receiving chemotherapy (HR = 0.384; 95% CI: 0.244 - 0.604, *P* < 0.001) are independent predictors of suicide among pancreatic cancer patients. We combined these results to identify the five most predictive factors, and based on these, we constructed a novel nomogram (Figure 4).

**Table 2:** Univariate and multivariate Cox analyses of suicide risk in the training cohort.

**Characteristic**	**Univariate analysis**			**Multivariate analysis**		
	**HR**	**95%CI**	***P***	**HR**	**95%CI**	***P***
**Year of diagnosis**						
2000–2006	Reference					
2007–2012	0.651	0.384–1.103	0.111			
2013–2020	0.684	0.424–1.104	0.120			
**Age at diagnosis, years**						
<65	Reference			Reference		
≥65	2.244	1.432–3.519	<0.001	2.295	1.450–3.630	<0.001
**Sex**						
Female	Reference			Reference		
Male	6.679	3.648–12.23	<0.001	7.902	4.256–14.673	<0.001
**Race**						
White	Reference					
Black	0.651	0.301–1.408	0.276			
Asian	0.589	0.238–1.451	0.250			
Other	1.102	0.153–7.918	0.923			
**Marital status**						
Married	Reference			Reference		
SDW	1.202	0.731–1.980	0.468	1.677	1.004–2.802	0.048
Unmarried	1.818	1.096–3.013	0.021	2.159	1.292–3.611	0.003
**Grade**						
Grade I/II	Reference					
Grade III/IV	0.609	0.266–1.401	0.244			
Unknown	1.479	0.932–2.349	0.097			
**Stage**						
Localized	Reference					
Regional	0.773	0.419–1.429	0.412			
Distant	1.553	0.863–2.794	0.142			
Unknown/unstaged	1.596	0.586–4.346	0.360			
**Primary site**						
Head	Reference					
Body/tail	1.162	0.724–1.862	0.533			
Other/unknown	1.322	0.802–2.180	0.274			
**Histological type**						
PDAC	Reference					
PCN	1.137	0.518–2.495	0.749			
pNET	0.707	0.363–1.372	0.305			
Unknown/Other	1.076	0.586–1.977	0.813			
**Surgery**						
No/Unknown	Reference			Reference		
Yes	0.406	0.255–0.646	<0.001	0.403	0.251–0.647	<0.001
**Radiotherapy**						
No/unknown	Reference			Reference		
Yes	0.431	0.217– 0.858	0.017	0.747	0.365–1.523	0.425
**Chemotherapy**						
No/Unknown	Reference			Reference		
Yes	0.371	0.241–0.569	<0.001	0.384	0.244–0.604	<0.001
**Annual household income**						
<$45,000	Reference					
$45,000–$74,999	0.944	0.377–2.359	0.901			
>$75,000	0.748	0.294–1.904	0.542			
**Residence**						
Metropolitan	Reference					
Non-metro/unknown	0.978	0.650–1.473	0.918			

SDW, Separated + Divorced + Widowed; HR, hazard ratio; CI, confidence interval.

### Establishment and validation of the nomogram

**Figure 5:**
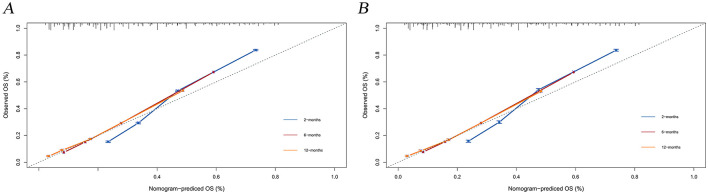
Calibration plots for the training and validation predictions. **(A)** presents the 2-month, 6-month, and 12-month endpoints for the training set; **(B)** presents the 2-month, 6-month, and 12-month endpoints for the validation set.

**Figure 6:**
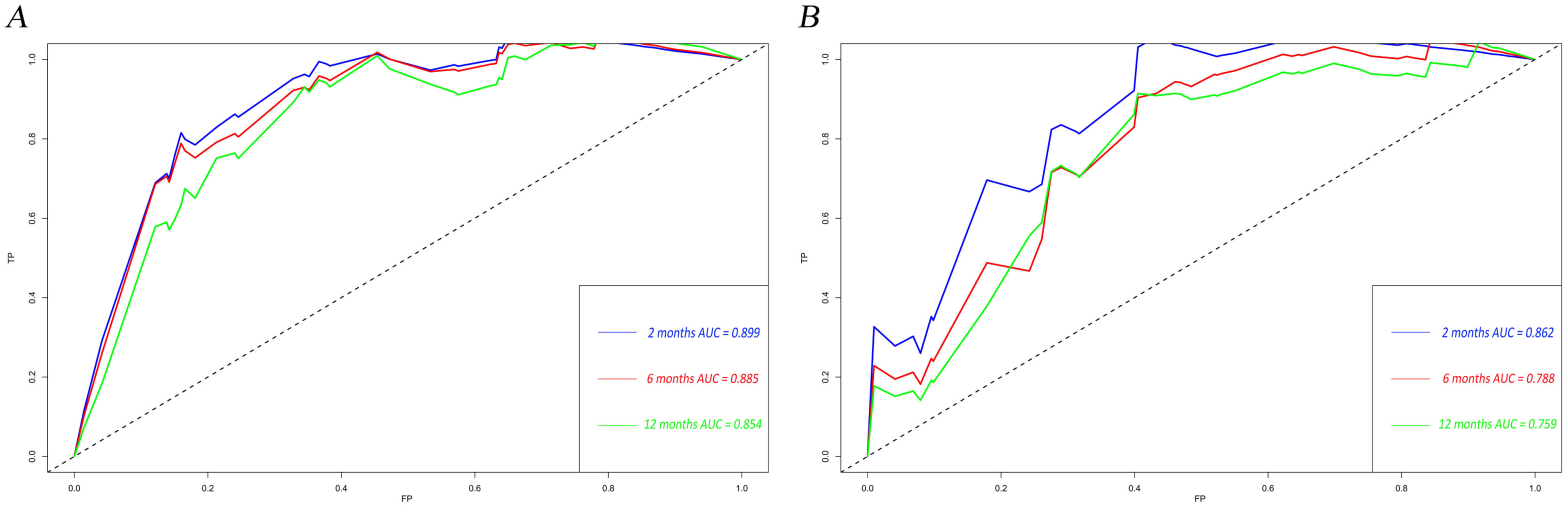
Time-dependent ROC curves for the nomogram model in the training set **(A)** and validation set **(B)**. TP, true positive; FP, false positive; AUC, area under the curve; ROC, receiver operating characteristic.

**Figure 7:**
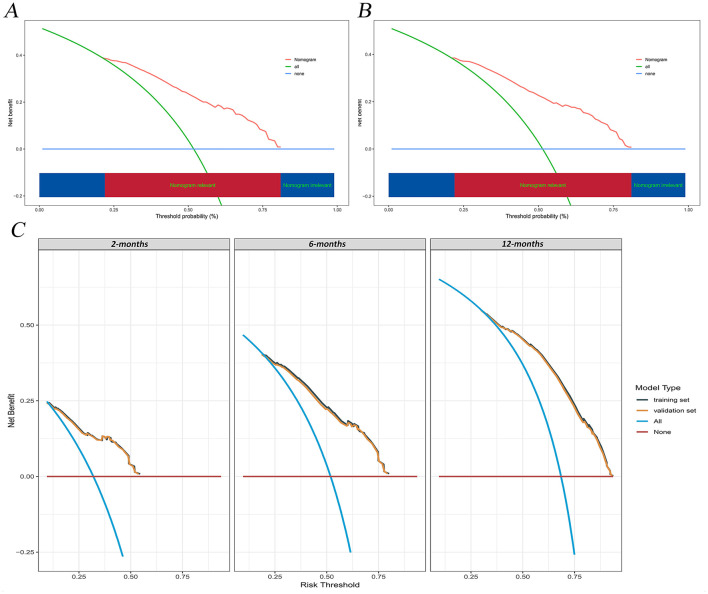
DCA of the nomogram model. **(A)** training set; **(B)** validation set; **(C)** Time-dependent DCA curves at 2, 6, and 12 months for the training and validation set. DCA, decision curve analysis.

**Figure 8:**
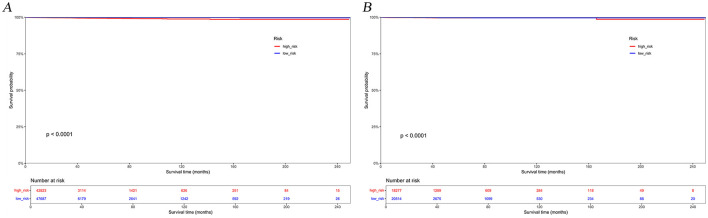
Kaplan-Meier survival curves for the training set **(A)** and validation set **(B)** based on risk groupings.

The calibration curves and time-dependent ROC curves were utilized to evaluate the predictive power of the nomogram model. The calibration curves at 2, 6, and 12 months for both the training and validation set demonstrated good predictive consistency (Figure 5). The horizontal axis represents the probability predicted by the nomogram, while the vertical axis depicts the actual proportion observed in the cohort. These curves visually illustrate the predictive performance of the nomogram at various points in time. The findings indicate that the predicted probability closely matched the actual observed proportion of deaths by suicide. The C-index for survival months in the training set and validation set were 0.872, 0.851, and 0.824, respectively. Additionally, ROC analysis confirmed the model's discriminative capability. Specifically, in the training set, the nomogram's AUC values at 2, 6, and 12 months were 0.899, 0.885, and 0.854, respectively (Figure 6A). At these same time points, the AUC values were 0.862, 0.788, and 0.759 in the validation set, respectively (Figure 6B). The DCA curves for the training set (Figure 7A) and validation set (Figure 7B) demonstrate the nomogram model's effective performance in clinical practice. Figure 7C further illustrates the DCA curves at 2, 6, and 12 months, indicating that the nomogram exhibits substantial clinical utility across different time points in both the training and validation cohorts. Over time, the performance of both sets remains consistent, suggesting that the model exhibits stable predictive capabilities. In addition, the K-M survival curves clearly demonstrated that patients in the high-risk group exhibited a higher suicide tendency compared to those in the low-risk group (*P* < 0.0001; Figures 8A, B).

## Discussion

Pancreatic cancer is an extremely aggressive malignancy of the digestive system, with an estimated 517,500 deaths worldwide each year (Siegel et al., 2024). Additionally, the study reveals that the suicide risk in pancreatic cancer patients is more than five times higher than in the general population, with a standardized mortality ratio (SMR) of 6.43 (Anderson et al., 2018; Ma et al., 2022). Nevertheless, predictive models for assessing suicide risk in pancreatic cancer patients remain lacking. Therefore, it is necessary to develop a new suicide risk assessment model for a specific population group (Fazel et al., 2019). The value of machine learning is especially evident when attempting to analyze datasets that are too large or complex for a human to handle (Greener et al., 2022). This study leveraged pancreatic cancer patient data from the SEER database, employing LASSO regression and Cox proportional hazards regression for variable selection to prevent overfitting, and subsequently developed a suicide risk prediction model. The nomogram calculates cumulative scores based on relevant patient variables to predict suicide risk in pancreatic cancer patients. Implementing this tool in clinical practice will enable a more precise approach to patient treatment plans, allowing physicians to identify suicide risk more quickly and intervene at the optimal time. According to our knowledge, our research is the first multicenter retrospective study to develop a nomogram model for predicting suicide risk in pancreatic cancer patients.

Our study indicates that the risk of suicide peaks at the initial cancer diagnosis but decreases over time (Figure 2). This suggests that the psychological and emotional stress linked to the diagnosis gradually decreases as time progresses. Male gender is suggested to be a risk factor for suicide in pancreatic cancer patients, according to our findings, which is consistent with previous studies (Zaorsky et al., 2019; Chen et al., 2021; Ravaioli et al., 2020). Men are more prone than women to impulsive, aggressive behavior and alcohol abuse, which can unintentionally lead them to focus more on immediate negative emotions, thereby intensifying psychological distress and increasing the risk of suicide (Gauthier et al., 2019; Spoletini et al., 2011). Additionally, for some men with poor prognoses or late-stage cancer, particularly those with a “Hemingway-type” personality (marked by independence, a strong emphasis on achievement, and stubbornness), suicide may be considered a rational and dignified choice when life expectancy is limited (Hietanen and Lönnqvist, 1991). Pancreatic cancer patients aged 65 and older have a higher tendency toward suicide compared to those under 65. Previous studies have demonstrated that the suicide rate among elderly cancer patients is markedly higher than in the general population (Mendes et al., 2021; Dormer et al., 2008). The elevated suicide risk in elderly patients may be linked to physical comorbidities, sleep disturbances, pain issues, cognitive impairments, and mental health conditions such as depression (Henson et al., 2019; Du et al., 2020). Furthermore, elderly cancer patients are more prone to experiencing depression, helplessness, and low self-esteem, which can lead to a decline in life satisfaction and, consequently, an increased risk of suicidal tendencies (Wu and Bond, 2006).

Pancreatic cancer is more prevalent in older adults, with the risk increasing with age, particularly in those aged 65 and above, which may be another reason for the elevated risk of suicide in this population. Individuals who are unmarried, separated, divorced, or widowed exhibit a higher propensity for suicide in comparison to their married counterparts. Prior research indicates that marriage mitigates the risk of suicide, presumably due to the emotional and social support it affords, which serves to alleviate social isolation (Osazuwa-Peters et al., 2018; Fukuchi et al., 2013). Our study shows that surgical intervention and chemotherapy serve as protective factors against suicide in individuals diagnosed with pancreatic cancer, with HRs of 0.384 (surgery, 95% CI: 0.251–0.647, *P* < 0.001) and 0.553 (chemotherapy, 95% CI: 0.244–0.604, *P* < 0.001), respectively. Similar findings have also been reported in several other studies (Ma et al., 2022; Shi et al., 2022). This observation may be attributed to continuous improvements in pancreatic cancer healthcare, particularly advancements in surgical techniques and the development of new chemotherapy agents and treatment strategies (Strobel et al., 2019; Neoptolemos et al., 2018). These advancements have collectively contributed to prolonging patient survival and mitigating suffering, thereby potentially decreasing the incidence of suicide among this patient population.

These findings underscore the importance of developing personalized support and prevention strategies based on cancer prognosis. Although palliative care for terminal cancer patients has gained attention, suicide risk has remained high over the past 25 years, indicating the need for greater focus on psychosocial support, pain management, and symptom control for cancer patients (Kumar et al., 2017). The transition to a biopsychosocial medical model underscores the critical importance of evaluating and managing suicide risk, as well as implementing suicide prevention strategies and psychological interventions for cancer patients (Fazel et al., 2020; Sullivan et al., 2018; Teno et al., 2013). Specifically, individuals diagnosed with pancreatic cancer generally encounter a notably poor prognosis. It is imperative to actively enhance treatment protocols and quality of life for these patients by offering improved medical care, comprehensive support systems, and specialized psychological counseling. Such measures are essential to mitigate the elevated risk of suicide among this population. Based on our nomogram, the treating oncologist or oncology nurse in routine practice enters five readily obtainable variables (age, sex, marital status, surgery, and chemotherapy) at the patient's first visit. The nomogram immediately produces an individual suicide-risk score that categorizes patients as high-risk or low-risk. High-risk patients are referred to psycho-oncology services after diagnosis, and the score is re-evaluated at key treatment milestones, such as after surgery or at the end of chemotherapy, allowing supportive care to be adjusted promptly. This efficient process facilitates critical early risk detection and targeted psychological support within the narrow therapeutic window of pancreatic cancer care.

However, we recognize several limitations in our study. Firstly, the data used in this study comes from a retrospective analysis of the SEER database, which could potentially introduce selection bias (Park et al., 2012). Although recent geopolitical developments have altered the access conditions for the SEER database, it is important to emphasize that our study utilized a complete dataset obtained prior to April 2025, thereby ensuring the integrity and validity of the data. Secondly, the constraints of the available data within the SEER database pose challenges in acquiring additional pertinent information, such as mental health issues (including depression and anxiety), comorbidities, and substance abuse. Lastly, it is imperative to recognize that our predictive model has been subjected solely to internal validation. External validation using independent cohorts is essential for evaluating its generalizability, as the absence of such validation may introduce inherent bias into our findings.

## Conclusion

Suicide constitutes a significant global public health concern, with patients diagnosed with pancreatic cancer exhibiting a heightened risk of suicide relative to the general population. Nevertheless, a considerable number of surgeons may not possess the requisite expertise to evaluate suicide risk subsequent to a cancer diagnosis, thereby leading to potential lapses in suicide prevention efforts. In this study, we employed a combination LASSO and Cox proportional hazards regression to identify five risk factors. Among these factors, being aged 65 years or older, male, unmarried, not having undergone surgical intervention, and not receiving chemotherapy are identified as high-risk factors for suicide in patients with pancreatic cancer. Subsequently, we developed a nomogram to predict suicide risk among pancreatic cancer patients. The nomogram exhibited robust performance with respect to discrimination, calibration, clinical applicability and generalization. This model enables clinicians to quickly identify high-risk individuals, allowing for timely and appropriate preventive measures to reduce unnecessary deaths and alleviate the burden of pancreatic cancer.

## Data Availability

The raw data supporting the conclusions of this article will be made available by the authors, without undue reservation.
